# Bimetallic copper palladium nanorods: plasmonic properties and palladium content effects†

**DOI:** 10.1039/d3na00523b

**Published:** 2023-10-24

**Authors:** Andrey Ten, Claire A. West, Soojin Jeong, Elizabeth R. Hopper, Yi Wang, Baixu Zhu, Quentin M. Ramasse, Xingchen Ye, Emilie Ringe

**Affiliations:** a Department of Materials Science and Metallurgy, University of Cambridge 27 Charles Babbage Road Cambridge CB3 0FS UK er407@cam.ac.uk; b Department of Earth Sciences, University of Cambridge Downing Street Cambridge CB2 3EQ UK; c Department of Chemistry, Indiana University 800 East Kirkwood Avenue Bloomington Indiana 47405 USA xingye@indiana.edu; d Department of Chemical Engineering and Biotechnology, University of Cambridge Philippa Fawcett Drive Cambridge CB3 0AS UK; e School of Chemical and Process Engineering, University of Leeds Leeds LS2 9JT UK; f School of Physics and Astronomy, University of Leeds Leeds LS2 9JS UK; g SuperSTEM, SciTech Daresbury Science and Innovation Campus Keckwick Lane Daresbury WA4 4AD UK

## Abstract

Cu is an inexpensive alternative plasmonic metal with optical behaviour comparable to Au but with much poorer environmental stability. Alloying with a more stable metal can improve stability and add functionality, with potential effects on the plasmonic properties. Here we investigate the plasmonic behaviour of Cu nanorods and Cu–CuPd nanorods containing up to 46 mass percent Pd. Monochromated scanning transmission electron microscopy electron energy-loss spectroscopy first reveals the strong length dependence of multiple plasmonic modes in Cu nanorods, where the plasmon peaks redshift and narrow with increasing length. Next, we observe an increased damping (and increased linewidth) with increasing Pd content, accompanied by minimal frequency shift. These results are corroborated by and expanded upon with numerical simulations using the electron-driven discrete dipole approximation. This study indicates that adding Pd to nanostructures of Cu is a promising method to expand the scope of their plasmonic applications.

## Abstract 2

Cu is an inexpensive alternative plasmonic metal with optical behaviour comparable to Au. Alloying with Pd imparts a catalytic surface, improves environmental stability, and retains plasmonic properties.

## Graphical abstract

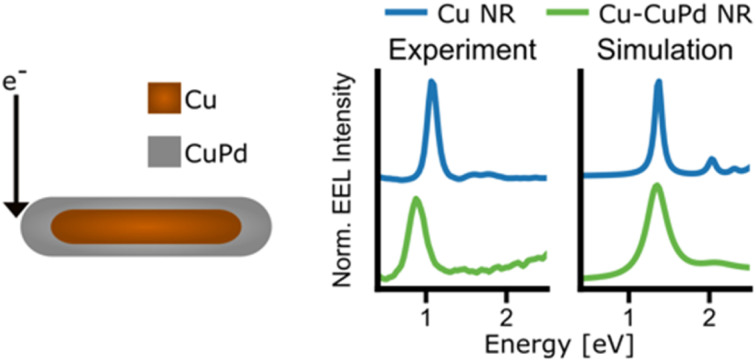

## Introduction

Nanoparticles (NPs) of some metals can sustain localised surface plasmon resonances (LSPRs), a light-driven coherent oscillation of conduction electrons leading to enhanced light absorption, scattering, and localised field at the surface of the NPs.^1–4^ The far-field properties of LSPRs are employed, *e.g.*, for refractive index sensing,^5,6^ while their near-field effects can be used to boost spectroscopic signals *via* surface-enhanced Raman spectroscopy (SERS)^7^ and metal-enhanced fluorescence (MEF).^8,9^ Furthermore, plasmonic processes are becoming increasingly important in photocatalysis^10^ and photothermal cancer treatment.^11^ The optimisation of such plasmonic applications involves tuning the LSPR peak position and width, achieved by adjusting the composition, size, and shape of the NP.^12,13^

The carrier densities and loss pathways vary widely between different plasmonic metals, and each material's performance varies across the electromagnetic spectrum. Ag for instance has low losses and a high quality factor across the ultraviolet, visible, and near-infrared (UV-Vis-NIR) ranges, while the interband transitions of Au prevent it from sustaining strong resonances in the UV and blue region of the visible.^14^ Au and Ag are ubiquitous in plasmonics,^15,16^ however new materials, including alternative metals such as Cu,^17,18^ Al,^19^ and Mg,^20^ offer compelling properties. Of these, Cu is a d^10^ metal, like Ag and Au, and has similar carrier density to its Group 11 neighbours,^21^ albeit suffering slightly more losses. Nonetheless, Cu nanostructures can sustain plasmons of quality comparable to those of Ag in the NIR,^18^ and Cu's abundance and relatively low cost makes it an attractive alternative to the other two coinage metals.^17,22,23^

Bimetallic NPs offer an opportunity to tune physical and chemical properties.^24^ Au and Ag, for example, are both good plasmonic materials, which when combined either as alloys or decorated structures also produce strong plasmon resonances with energies in between those of the constituent metals,^25–27^ for application in sensing^28–30^ and photothermal^31,32^ effects. Bimetallic NPs lead to more than a simple mixture of dielectric functions: they can also deliver additional functionality and/or improved stability.

To add functionality, a NP can be alloyed or decorated with a catalytically active metal, for instance Pd.^33^ However, dielectric function mixing is also occurring, such that the plasmonic properties of the bimetallic structure may suffer from the addition of a poor plasmonic metal such as Pd, in which interband transitions are significant across the UV-Vis-NIR.^34^ Despite the expected damping, plasmon resonances have been demonstrated in Au NPs containing Pd,^26,35^ and subsequently alloying and decoration have been successful in imparting catalytic properties to plasmonic structures based on Au,^36,37^ Ag,^38,39^ Cu,^40–42^ Al,^43,44^ and Mg,^45^ to name a few.

Alloying and core–shell architectures can also be used to enhance the stability of oxidation-prone plasmonic metals. This is of particular interest for Cu, for which the native oxide layer is neither self-limiting^46^ nor protective towards reaction with air or water.^23^ Alloying Cu with Ga,^46^ Au,^47^ and Ag^48^ has been reported to reduce the rate of oxidation, and a similar effect may be expected from the catalytically active but poorly plasmonic Pd. However, this potential increase in stability is not impact-free to the other properties, and it is important to understand and quantify the trade-offs of incorporating catalytic materials into plasmonic nanoparticles.

Plasmonic properties of NPs can be probed with sub-angstrom spatial resolution using scanning transmission electron microscopy electron energy-loss spectroscopy (STEM-EELS). In the early days, the low-loss region where LSPRs with energies in the visible range or lower were challenging to observe due to the broad zero-loss peak (ZLP) creating a high background and leading to peak broadening. However, the advancements of electron microscopes, including the development of monochromators, has led to narrower ZLPs.^49^ These new capabilities now allow low-energy studies, such as those in the range of energies where phonon modes,^50–52^ molecular vibrations,^53^ and low energy LSP modes can be observed.

Here, we synthesise Cu nanorods (NRs) and bimetallic Cu NRs coated with a CuPd alloy shell and characterise their plasmonic character as a function of increasing Pd content. The optical response is experimentally obtained with monochromated STEM-EELS and simulated using the electron-driven discrete dipole approximation (e-DDA).^54^ We first characterise the plasmonic response of Cu NRs of lengths 23–270 nm using STEM-EELS. We find that Cu NRs support plasmon modes in the Vis-NIR wavelengths. We then compare this result to the plasmonic properties of bimetallic Cu–CuPd NRs with different amounts of Pd. Cu–CuPd NRs are damped compared to Cu NRs but their plasmonic character remains up to at least 46 mass percent of Pd. We find that the dimensions of NRs influence the plasmon energy more significantly than Pd content. We conclude that the Cu–CuPd NRs synthesised in this study are viable options to modify Cu nanostructures without markedly diminishing their plasmonic properties, which motivates these particles as candidates for plasmon-assisted photocatalysis.

## Methods

### Chemicals

Hydrogen tetrachloroaurate trihydrate (HAuCl_4_·3H_2_O, ≥99.9% trace metals basis), copper(ii) chloride dihydrate (CuCl_2_·2H_2_O, 99.999%), oleylamine (70%), borane *tert*-butylamine complex (97%), palladium(ii) chloride (PdCl_2_, 59% Pd), acetone (99.5%), anhydrous toluene (99.8%) and isopropanol (99.5%) were purchased from Sigma Aldrich. Oleylamine (70%) from Sigma Aldrich (OLAM-SA) was used as received without further purification. Oleylamine (50%) was purchased from TCI America (OLAM-TCI) and was pre-dried under vacuum at 100 °C for 4 h and stored inside a N_2_-filled glovebox before use. All glassware was cleaned with aqua regia (a mixture of HCl and HNO_3_ in 3 : 1 volume ratio), followed by rinsing with water and drying before use.

#### Synthesis of Au nanocrystal seeds

6.5 nm Au nanocrystal seeds were synthesised using our previously reported method.^17,55^ In a typical synthesis, 10 mL of OLAM-TCI was placed in 50 mL three-neck round-bottom flask, followed by vacuum degassing for 30 min at room temperature. After refilling with N_2_, 10 mL of toluene and 0.25 mmol HAuCl_4_·3H_2_O were mixed with dried OLAM. The mixture was purged with flowing N_2_ for 10 min, then cooled to 15 °C using an ice bath. Subsequently, the mixture of 0.25 mmol borane *tert*-butylamine complex, 1 mL OLAM-TCI, and 1 mL anhydrous toluene was swiftly injected to the reaction solution under stirring, followed by reacting for an hour at 15 °C. Au nanocrystal seeds were isolated by precipitation with 60 mL acetone, followed by centrifugation at 6000 rpm for 5 min. The precipitates were re-dispersed in anhydrous toluene to attain optical density (O. D.) of 40 at LSPR peak wavelength (525.3 nm). OLAM-SA can be used in place of OLAM-TCI, but the latter produced more uniform Au nanocrystal seeds in terms of size and shape.

#### Synthesis of Cu NRs

Cu NRs were synthesised following our previously reported seed-mediated growth.^17,55^ In a typical reaction, 0.5 mmol CuCl_2_·2H_2_O and 10 mL of OLAM-SA were charged in the 50 mL three-neck round-bottom flask, and the mixture was dried using 3 cycles of vacuum degassing followed by N_2_ flushing. The mixture was heated and kept at 80 °C for 1 h to fully dissolve CuCl_2_ precursors, resulting in a blue solution. The solution was then heated to 180 °C, and a set volume of the Au seeds solution (O. D. = 40) was injected under stirring, after which it was left to react at 180 °C for 1 h. After cooling to room temperature, Cu NRs were collected through precipitation with 30 mL of isopropanol, followed by centrifugation at 4500 rpm for 3 min. The resultant pellets were re-dispersed in anhydrous toluene with 20 s of ultrasonication, then stored in a N_2_-filled glovebox. Cu NRs with average lengths of 28, 35, 70, 100, 120, 140, and 240 nm were synthesised by injecting 0.40, 0.35, 0.25, 0.20, 0.15, 0.10, and 0.05 mL of Au seeds solution (O. D. = 40), respectively. OLAM-TCI can be used in place of OLAM-SA, but the latter produced more uniform Cu NRs in terms of size and shape.

#### Synthesis of Cu–CuPd NRs

Typically, 0.5 mmol of CuCl_2_·2H_2_O and 10 mL of OLAM-SA were placed in a 50 mL three-neck round-bottom flask. The mixture was dried through 3 cycles of vacuum degassing followed by N_2_ flushing. The mixture was then heated and kept at 80 °C for 1 h, after which it was further heated to 180 °C. Subsequently, 0.20 mL of the Au seeds solution (O. D. = 40) was swiftly injected at 180 °C under stirring. A set amount of PdCl_2_ and 1 mL of OLAM-SA was completely mixed by stirring in a 4 mL scintillation vial at 80 °C. The resulting transparent Pd-OLAM solution was slowly injected to the reaction mixture at 25 μL min^−1^, 15 min after the addition of the Au seeds solution. The total reaction time at 180 °C was 1 h. Cu–CuPd NRs were isolated *via* flocculation with 30 mL isopropanol, followed by centrifugation at 3000 rpm for 3 min. The resulting pellets were re-dispersed in anhydrous toluene with 20 s of ultrasonication, then stored in N_2_-filled glovebox. Cu–CuPd NRs with 22, 42, and 46 mass percent of Pd were synthesised by injecting 0.05, 0.10, and 0.20 mmol PdCl_2_, respectively. OLAM-TCI can be used in place of OLAM-SA, but the latter produced more uniform Cu–CuPd NRs in terms of size and shape.

#### Polymer functionalisation of Cu and Cu–CuPd NRs

NRs were grafted with polystyrene-pentaethylenehexamine (PS-PEHA) to improve colloidal stability. PS-PEHA was synthesised in-house following our previously reported method.^56^ In a typical reaction, 0.5 mL of NR solution (5 mg mL^−1^ in anhydrous toluene) was added to the mixture of 20 mg of 5.6 k PS-PEHA and 1.6 mL of anhydrous tetrahydrofuran. The solution was sonicated for 10 s and was left undisturbed for 12 h inside a N_2_-filled glovebox. Afterwards, NRs were retrieved *via* precipitation with anhydrous heptane, followed by centrifugation at 3000 rpm for 3 min. The precipitates were redispersed in anhydrous toluene or tetrachloroethylene for TEM or UV-Vis-NIR measurements, respectively.

#### Characterisation of Cu and Cu–CuPd NRs

TEM images were obtained on a JEOL JEM 1400 plus microscope equipped with a LaB_6_ filament operating at 120 kV. TEM samples were prepared by drop-casting ∼10 μL of sample solution onto a 300-mesh carbon-coated nickel grid (Ted Pella).

UV-Vis-NIR spectroscopy was performed using a Varian Cary 5000 UV-Vis-NIR spectrophotometer (Agilent).

STEM-energy dispersive X-ray spectroscopy (STEM-EDS) measurements of Cu–CuPd NRs drop cast on a 10 nm thick Si_3_N_4_ membrane were acquired at 200 kV on a FEI Osiris STEM equipped with a Bruker Super-X quadruple EDS detector. EDS spectral data was processed using an open-source software, Hyperspy.^57^ The dataset was first summed across the acquired region to fit a background. For each of the K_α_ lines of C (0.28 keV), Cu (8.05 keV), N (0.39 keV), O (0.52 keV) and Si (1.74 keV), and L_α_ lines of Au (9.71 keV) and Pd (2.84 keV), a linear background was fitted between regions below and above the peak. Using this background, the X-ray lines were integrated for each element across the acquired region where the width of integration was set to the extended energy resolution of Mn K_α_ from the detector. The atomic composition was then quantified using the Cliff–Lorimer ratio method.

Low-loss STEM-EEL spectra of Cu and Cu–CuPd NRs drop cast on a 10 nm thick Si_3_N_4_ membrane (Simpore Inc) were obtained on a Nion UltraSTEM™ 100 MC High Energy Resolution Monochromated EELS-STEM (HERMES) microscope, a dedicated STEM equipped with a cold field electron emitter, and Nion's ultrahigh resolution ground-potential monochromator. The microscope was operated at 60 kV, and the probe forming optics were configured to provide an electron probe of 31 mrad convergence semi-angle and a probe current of 50 pA before closing the monochromator slit, corresponding to a probe size of approximately 1 Å. EEL spectra were recorded on a Nion Iris spectrometer, equipped with a Dectris ELA hybrid pixel detector camera. A 44 mrad semi-angle EELS entrance aperture was used for all data. The system's monochromator slit was closed as required by the desired energy resolution, resulting here in a 100 meV full width at half-maximum of the zero-loss peak, FWHM ZLP, and 5–7 pA current. The Nion's high-angle annular dark field (HAADF) imaging detector was set at an angular range of 90–195 mrad. EEL spectral data was processed using an open-source software, Hyperspy.^57^ For every spectrum, the ZLP was used to align the energy axis with subpixel accuracy. The spectrum was then cropped from 0.15 eV to 4.00 eV, after which X-ray spikes were removed, minimum intensity shifted to 0, and plasmon modes extracted using non-negative matrix factorisation (NMF).^58–61^ The number of NMF components was optimised for individual NRs, by selecting the largest value which did not cause the duplicate factorisation of identical modes. A Lorentzian line shape was fitted to individual NMF factors to obtain energy and FWHM values. The dimensions of NRs were recorded from HAADF-STEM images by measuring the tip-to-tip distance for length and side-to-side distance for width.

Inductive coupled plasma mass spectroscopy (ICP-MS) measurements were performed using a PerkinElmer Nexion 350D quadrupole-based Inductively Coupled Plasma-Mass Spectrometer. Samples were digested in an aqueous matrix with 10% v/v of ultra-pure metal trace nitric acid (max 10 ppt metal traces) and diluted to ∼10 ppb (μg L^−1^).

Powder X-ray diffraction (XRD) patterns were collected on a PANalytical Empyrean X-ray diffractometer with a Cu source operated at 40 kV and 35 mA. Samples for XRD measurements were prepared by drop-casting *ca.* 0.5 mL of colloidal NR solutions (20 mg mL^−1^ in chloroform) onto single-crystalline Si substrates.

#### Simulation of Cu and Cu–CuPd NRs

The EEL point spectra and spectrum image slices were simulated using the Maxwell solver e-DDA,^54^ which uses the discrete dipole approximation with an electron driving force. The Cu and Cu–CuPd NR lengths and widths were determined from the HAADF-STEM images, as previously described. The thicknesses of the NRs were assumed to be equal to the widths. The Cu NRs were simulated using a Drude model fit to Johnson and Christy data,^21^ to approximate the dielectric function of the NRs. The NRs were encased in a 1 nm Cu_2_O shell (dielectric data from Palik^62^), to model the 1 nm oxide layer, a thickness previously reported.^17^ The NRs were simulated in an infinite background of *n* = 1.3 to approximate the image-induced redshift caused by the Si_3_N_4_ substrate. The Cu–CuPd NRs were composed of a Cu core surrounded by a shell of CuPd alloy at 50 : 50 atomic percent. We used the dielectric function calculated by Rahm *et al.*,^63^ and the background was set to *n* = 1.3. Note that the CuPd dielectric function was calculated in ref. 63 to model a fully disordered alloy with an atomic ratio of precisely 50 : 50. In practice, the CuPd shell is unlikely to be exactly 50 : 50 nor perfectly homogeneously disordered. Given that the dielectric function changes with respect to alloy ratio and ordering, the approximation to use the aforementioned dielectric function may impact the agreement between experiment and simulation. All LSPR energies and FWHM values were determined by fitting a Lorentzian line shape to the simulated data.

## Results and discussion

**Fig. 1:**
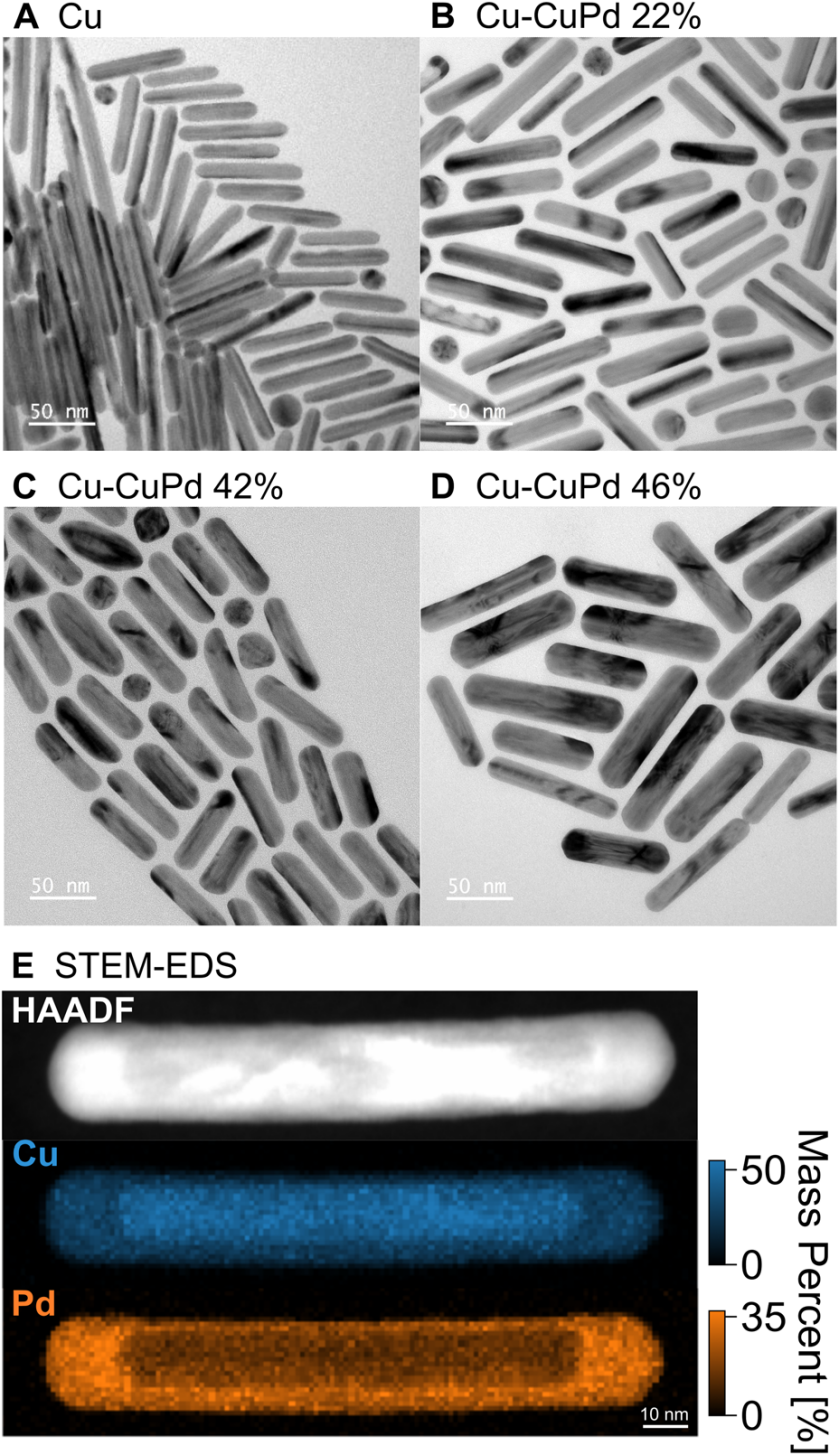
Shape and composition of Cu and Cu–CuPd NRs. TEM image of representative (A) Cu NRs and Cu–CuPd NRs with varying concentrations of Pd precursor resulting in NRs with Pd mass percent of (B) 22, (C) 42, and (D) 46%. (E) HAADF signal and STEM-EDS elemental maps for Cu and Pd in a Cu–CuPd NR from the batch containing 46% Pd by mass.

Cu NRs were synthesised using a heterometallic seed-mediated growth approach in six batches of varying concentrations of Au seeds,^17^ which provided NR lengths from 20 nm to 270 nm, with an average NR width of 12 nm. A representative TEM image from one of the synthesis batches of Cu NRs (0.25 mL of Au seeds solution, NRs ∼70 nm in length), is shown in Fig. 1A. The Cu NRs possess a thin oxide shell approximately 1 nm thick (Fig. S1†) and a residual amount of Au from the Au nuclei used, as previously reported in Jeong *et al.*^17^ The NRs display contrast attributed to twinning: they are pentatwinned as previously described.^17^ The LSPR peak in the UV-Vis-NIR extinction spectra redshifted and broadened with increased NR length (Fig. S2A†), as also reported previously.^17^

The Cu–CuPd bimetallic NRs were synthesised by adding varying amounts of Pd precursor during the synthesis of the aforementioned Cu NRs using the procedure which would otherwise produce 100 nm × 12 nm Cu NRs. Adding increasing amounts of precursor (0.05 mmol, 0.1 mmol, and 0.2 mmol of PdCl_2_) led to sets of Cu–CuPd bimetallic NRs of increasing Pd content (Fig. 1 and S3†), with Pd mass percent of 22, 42, and 46%, as determined by ICP-MS. The Cu–CuPd NRs formed a core–shell geometry, where the core is Cu and the shell is a 50 : 50 atomic percentage alloy of Cu and Pd, as confirmed by STEM-EDS (Fig. 1E and S4†). Cu and Pd indeed form predominantly CuPd and Cu_3_Pd at the synthesis temperature, such that in the current Pd-rich conditions the stoichiometric CuPd is obtained.^64^ The CuPd alloy shell grew over the five-fold twinned Cu core, leading to the geometry observed in the TEM images of Fig. 1 and S3.† The Cu and Pd atoms in the alloy shell were distributed randomly, as evident by the disordered A1 phase of CuPd observed in XRD patterns of Cu–CuPd NRs (Fig. S5†).^65^ The absence of the (100) reflection indicative of the B2 phase further confirms the disordered nature of the alloy shell.^65,66^

The Pd precursor concentration influences the thickness of the CuPd alloy shell, with on average thicker shells forming with greater amounts of Pd. The average width of the 22, 42, and 46% Cu–CuPd NRs was 14 ± 2, 16 ± 2, and 22 ± 4 nm, respectively, while their average length was 66 ± 12, 62 ± 7, and 89 ± 14 nm, respectively (100 NRs sampled for each, see Fig. S3†). Note that there remained less than 2 mass percent of Au near the tip of the Cu core from the synthesis of Cu NRs (Fig. S6†) and no appreciable oxide layer on the CuPd shell was observed based on STEM-EDS maps (Fig. S4†). Cu–CuPd NRs with increasing Pd content had a broader peak in bulk UV-Vis-NIR extinction spectra (Fig. S2B†), meanwhile no clear trend was observed for the peak energy, provoking the need for a rod-by-rod analysis using STEM-EELS.

**Fig. 2:**
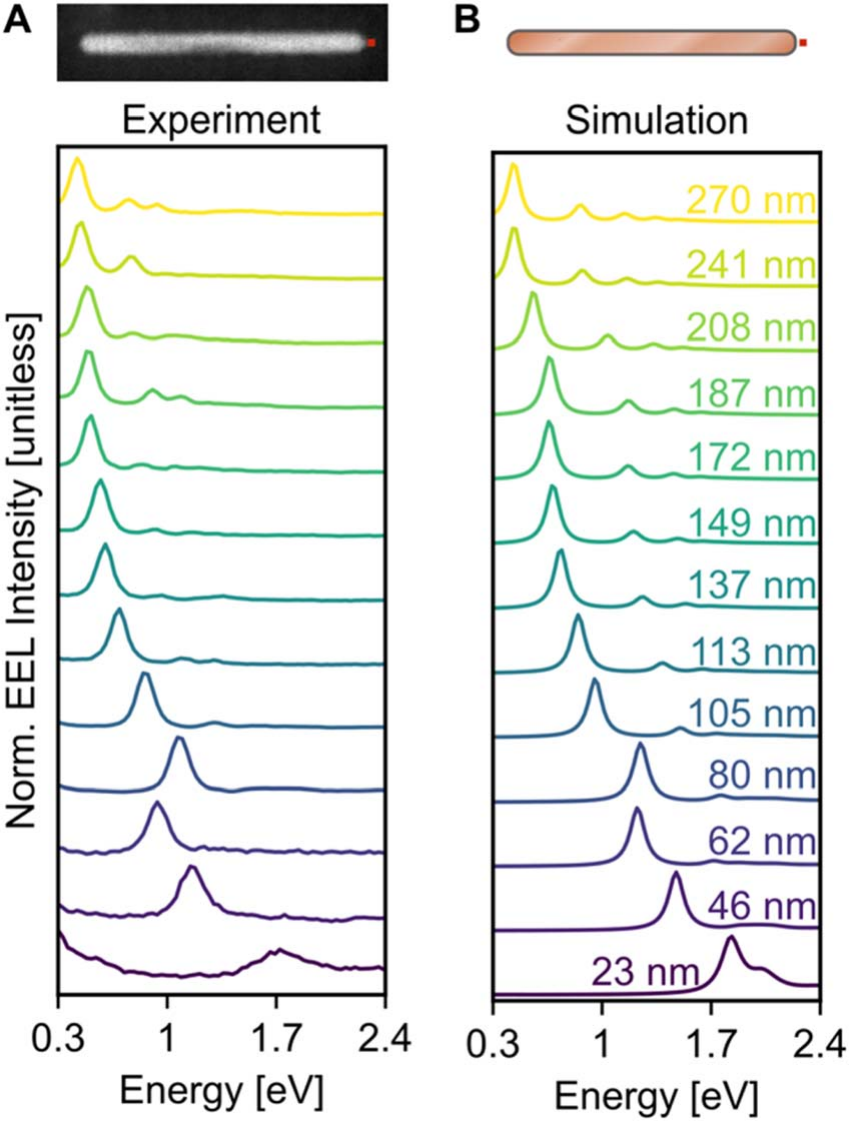
Size dependent behaviour of the EEL response of individual NRs. (A) Experimental and (B) simulated normalised EEL point spectra of Cu NRs of lengths ranging from 23 nm (purple) to 270 nm (yellow), as indicated in (B). The experimental spectra were summed over 3 × 3 pixels in the region equivalent to that indicated by the red square in the HAADF-STEM image in (A), while the simulated spectra were calculated at an equivalent position, *e.g.*, 4 nm offset from the NR tip.

Electron excitation spectra of Cu NRs displayed a low energy dipolar LSPR as well as a series of higher order modes. These LSPRs were observed experimentally using monochromated STEM-EELS, examples of which are reported in Fig. 2 and S7.† The STEM-EEL spectra displayed in Fig. 2A were obtained with the electron beam positioned at the tip of the NR, leading to a strong excitation of the longitudinal dipole (lowest energy) mode, and several higher energy modes. As expected, the prominent longitudinal modes redshift with increasing NR length; for instance, the longitudinal dipole LSPR shifted from 1.70 eV for the 23 nm NR to 0.42 eV for the 270 nm NR. The 80 nm NR is wider than average (14 nm width), causing its LSPRs to blueshift, as expected. Numerical simulations exactly matching the NR lengths and widths (Fig. 2B) confirmed the experimentally observed trends, including the longitudinal dipole LSPR shift, numerically from 1.83 eV (23 nm NR) to 0.45 eV (270 nm NR), and the out-of-trend behaviour of the wide 80 nm NR. We mitigated the effects of the substrate as much and as simply as possible by enclosing the NRs in a uniform background of refractive index 1.3. However there remain differences in the resonance energy and linewidth of the LSP modes attributed to using the aforementioned approximation, and the idealised computed shapes compared to the reality of porous oxide shells and imperfect growth geometries.

**Fig. 3:**
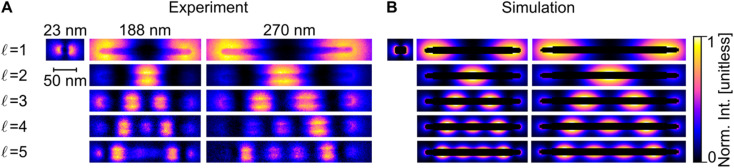
Longitudinal LSP mode excitation distribution in Cu NRs. (A) STEM-EELS NMF spatial loadings (experiment) and (B) EEL probability maps (simulation) at different energies for the 
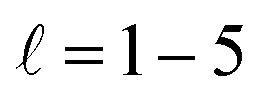
 LSP modes of three representative Cu NRs of labelled length and width of 12–13 nm.

Blind source separation, a type of machine learning,^67^ was used to analyse the experimental STEM-EEL spectrum images, revealing a rich set of resonant modes with varying spatial distribution. Specifically, we used non-negative matrix factorisation (NMF), as described in Nicoletti *et al.*,^61^ to globally fit each spectrum image, leading to the generation of spectral components (Fig. S8†) and corresponding spatial loadings (Fig. 3A). NMF imposes a non-negative constraint on the output, but no constraints on the spectral or spatial distribution; it is therefore reassuring to obtain mostly well-separated, realistic Lorentzian-shaped peaks as the spectral components (Fig. S8 and S9†). The spatial distribution of the magnitude of the excitation of these spectral components, corresponding to LSP modes, display an increasing number of longitudinal nodes for an increasing mode energy. These modes are denoted by 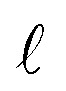, going up to 5 in Fig. 3. The number of resolvable modes with NMF was limited by mode overlap at high energy; we could extract up to seven modes for long rods (Fig. S10†) but only one for the shortest rod (Fig. 3 and S8†). The generation of images across an energy range in the STEM-EELS data set, mimicking energy filtered TEM (EFTEM), is an alternative approach to analyse this experimental data; results are shown in Fig. S11† and do not differ significantly from the NMF spatial distribution except for the inability of the EFTEM-like approach to extract spectral information. Excellent spatial agreement is achieved between experimental (Fig. 3A) and numerical (Fig. 3B) results.

**Fig. 4:**
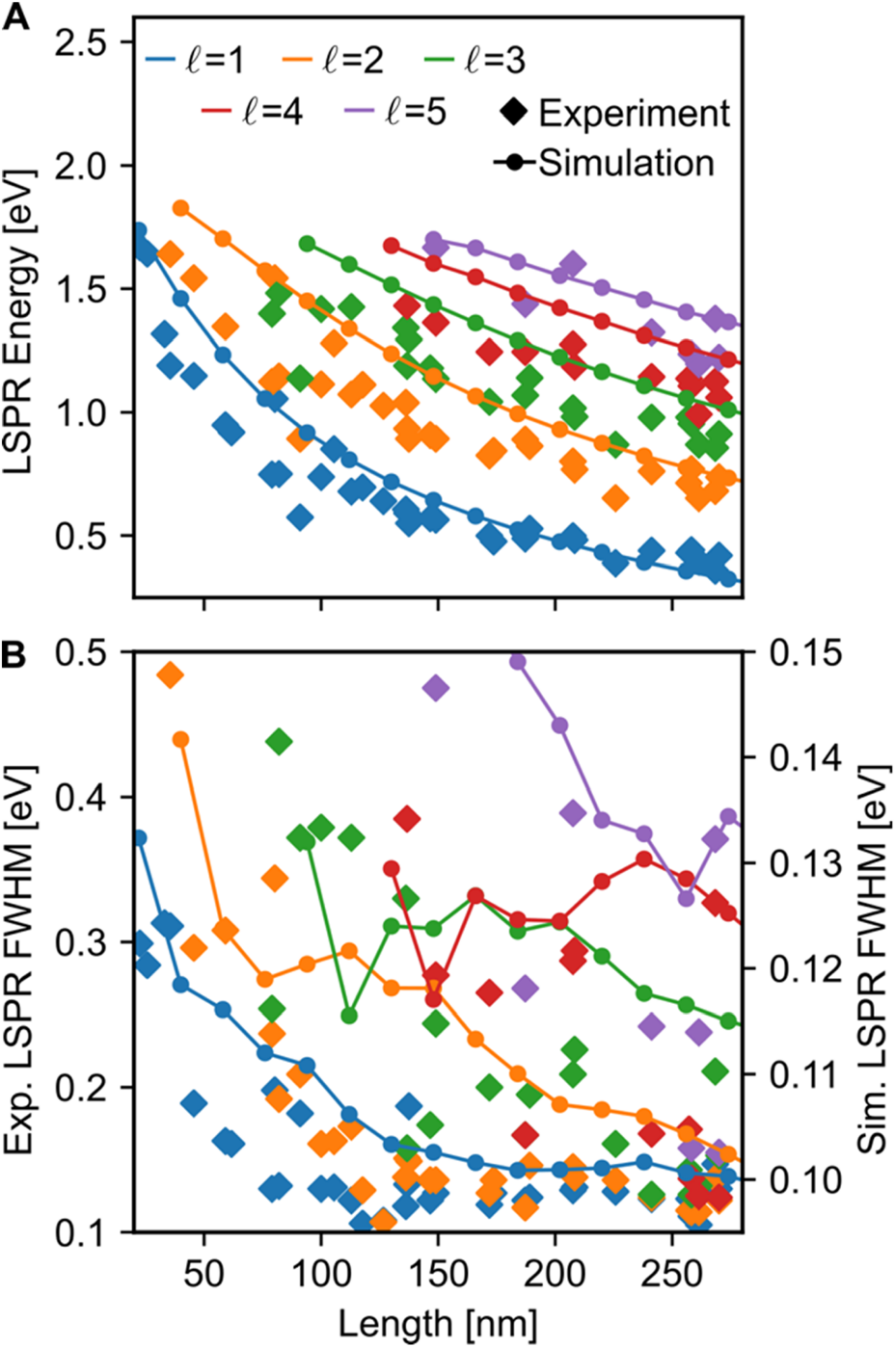
Length effect on the resonances of Cu NRs. (A) Energies and (B) FWHMs of the color-coded longitudinal LSPRs. The diamonds are experimental values and the connected dots are numerical simulations for 12 nm wide NRs. In (B), the left *y*-axis corresponds to the experimental data, and the right *y*-axis corresponds to the simulated data.

Both the LSPR energy and linewidth vary with Cu NR size, as revealed by fitting 35 STEM-EEL spectrum images (Fig. 4) from individual NRs (Table S1†). As the NR length increases, the plasmon energy of all LSP modes redshifts (Fig. 4A), as expected, and consistent with previous publications.^3,68,69^ For instance, the 
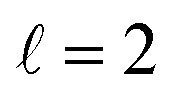
 mode shifted from 1.64 eV for a 35 nm NR to 0.65 eV for a 260 nm NR. The rate of change in plasmon energy decreased with increasing length, particularly for lower order modes. Similar to the LSPR energies, the full-width at half-maximum (FWHM) of the modes decreased with increasing NR length, and increased with increasing mode order (Fig. 4B). The former suggests that radiative damping from the increased volume in the Cu NRs is insignificant for the size range studied here (width ∼12 nm). LSPRs of NPs greater than 20 nm in diameter are dominated by radiative damping where FWHM increases with increasing diameter.^70,71^ On the other hand, NPs smaller than 10 nm experience strong electron-surface scattering, leading to an increase in FWHM with decreased size.^69,70^ Here, Cu NRs likely partially experience this effect, however the dominant effect is likely the decreased rate of non-radiative decay from interband transitions at lower LSPR energies, as suggested for Au NRs of similar widths.^72,73^ Cu's interband transition become important at ∼2.1 eV,^74^ and modes with energies approaching 2 eV suffer most from this damping mechanism. Indeed, the LSPR FWHM of Cu NRs shows a clear increasing trend with increasing LSPR energy (Fig. S12†), and this has also been observed with LSPRs below 2 eV of other Cu nanostructures.^18^ The increase in FWHM with increasing mode order can equally be explained by higher order modes appearing at higher energies closer to Cu's interband transition, rather than being intrinsic to increasing mode order. The experimental results were corroborated by numerical simulations (lines overlay in Fig. 4 and S12†) on Cu NRs of varying lengths, for which, to separate out width effects, a uniform width of 12 nm was used. The trends of decreasing energy and FWHM with increasing length are well reproduced numerically; the only mismatch is in the absolute value of the FWHM and magnitude of its variation, effects due to the lack of substrate in the numerical simulations.^2^

**Fig. 5:**
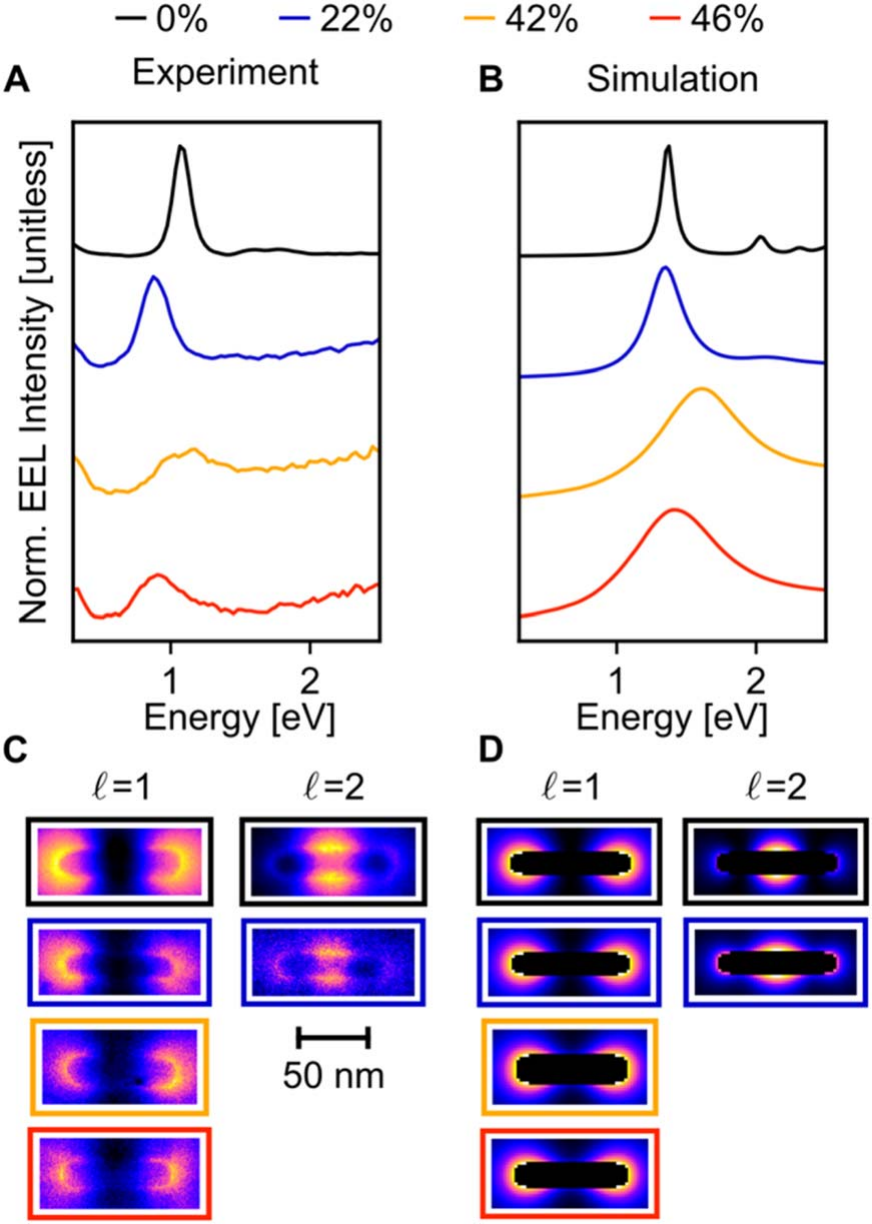
LSPR behaviour of Cu and Cu–CuPd NRs of similar sizes. The experimental spectra (A) were summed over 5 × 5 pixels at the tip of the NR, and the simulated spectra (B) were calculated at equivalent positions. The Cu NR (80 nm × 14 nm, black trace) supports three NMF resolvable LSP modes (up to 
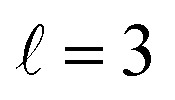
), but only two are shown. (C) NMF spatial loadings and (D) simulated EEL maps at the LSPR energies of the full Cu and Cu–CuPd NRs. Two LSP modes were resolvable for the Cu–CuPd NR of 22% Pd (77 nm × 13 nm, blue trace); while only one was resolvable for the Cu–CuPd NRs of 42% Pd (70 nm × 18 nm, yellow trace) and 46% Pd (75 nm × 16 nm, red trace). The colour of the frames in (C and D) correspond to their respective spectra in (A and B).

When compared to Cu NRs, Cu NRs coated with a CuPd shell display a significant decrease in the EEL signal amplitude and an increase in mode linewidth, indicating damping. The increase in damping with an increase in Pd content is particularly obvious in the 
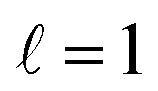
 mode, as shown in the representative point spectra extracted from STEM-EELS data sets on 24 NRs (Table S2†) of three different Pd compositions (Fig. 5A). The EEL signal amplitude relative to the ZLP was also on average 33% lower (Table S3†) and the NMF-extracted EEL probability distribution appeared more delocalised (Fig. 5C) for Cu–CuPd NRs than for Cu NRs. Analysis of this data presented some difficulties, however: the decreased amplitude and increased damping led to a lowering of the number of modes extractable in NMF, with the 
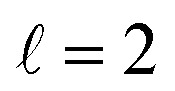
 mode being the highest order mode for Cu–CuPd NRs while Cu NRs of equivalent lengths were decomposed up to the 
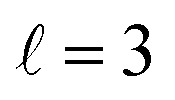
 mode. Also, the accumulation of carbon contamination during acquisition limited our ability to use the entire data set and extract spectral factors, as discussed in the ESI,† such that the LSPR energies and FWHMs subsequently presented were extracted from the half of the NRs with the least contamination (*i.e.*, the half acquired first). Companion simulations of the EEL spectra (Fig. 5B) and EEL maps (Fig. 5D) of Cu–CuPd NRs with a Cu core and a 50 : 50 atomic percent CuPd alloy shell at identical lengths and widths corresponding to experiment validate these trends. Qualitatively, the numerics match well to the experiment, except for a substrate-induced redshift which was not included in simulations, and the broader experimental results likely due to contamination.

**Fig. 6:**
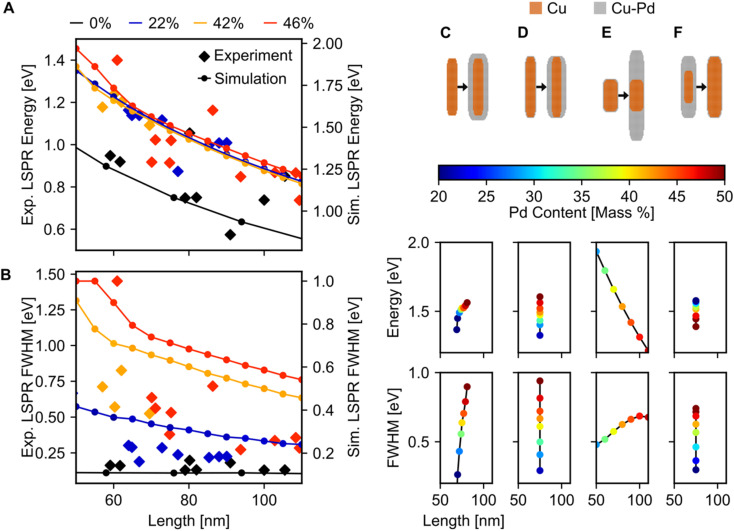
Effect of Pd content on LSPR energy and FWHM. Dipolar LSPR (A) energy and (B) FWHM for Cu and Cu–CuPd NRs of varying lengths and Pd content, left *y*-axis experiment, right *y*-axis simulation. (C–F) Simulations of four different geometries of core–shell growth. Each dot is a separate NR geometry, determined by interpolating between the two indicated schematics above, where marker colour indicates the percentage of Pd in each NR.

Fitting of the LSP modes revealed a quantitative increase in FWHM with Pd content, but no appreciable shift in the dipolar LSPR energy (Fig. 6A and B). The FWHM indeed increased up to a factor of six when Pd content was increased from 0 to 46%, although some of this increase may be due to the increased carbon contamination observed for all Cu–CuPd NRs compared to Cu NRs. Meanwhile, the LSPR energies of the Cu–CuPd NRs with three different Pd concentrations blueshifted from that of the Cu NRs, and all Cu–CuPd NRs followed the same trend with respect to NR length. Both trends were well matched numerically, except for the aforementioned substrate-induced redshift and contamination-induced broadening. Importantly, the qualitative trends are identical in both simulations and experiment, *i.e.*, LSPR energies are higher for NRs which contain Pd compared to those that do not, and LSPR linewidth of the NRs increases with increasing Pd content.

Most of the experimental trends discussed above are also convoluted with the shape change occurring between batches of different Pd content. Numerical results indicated that for NRs with Pd content from 20–50%, any geometry of addition of Pd will increase the FWHM, while the trend for LSPR energy shift varies (Fig. 6C–F), rationalising experimental results. This effect was investigated by simulating four different geometries of core–shell growth: fixed core with uniform shell growth (Fig. 6C), fixed core with shell growth only along the width (Fig. 6D), fixed core with shell growth only along the length (Fig. 6E), and uniform core growth with fixed total dimensions (Fig. 6F). When the shell expanded in all directions, the dipolar LSPR energy and LSPR FWHM increased with increasing Pd content (Fig. 6C), as observed experimentally. The same trend was observed for NRs with expanding widths (Fig. 6D), however, increasing the length led to a LSPR energy decrease with increasing Pd content (Fig. 6E). This trend can be attributed to retardation effects. Similarly, an increasing FWHM and decreasing LSPR energy occurred when the Cu : CuPd ratio was altered towards more CuPd (Fig. 6F). These results support our observation of increased FWHM due to increasing Pd content and provide an explanation for the lack of experimental LSPR energy shift.

## Conclusion

In this study, we analysed the increase in damping of LSP modes resulting from alloying Cu NRs with Pd, characterised using STEM-EELS and e-DDA simulations. Cu NRs supported longitudinal LSP modes which redshifted with increasing NR length, as expected. Addition of a Pd precursor to the reaction mixture resulted in the formation of a CuPd alloy shell around the Cu NR. STEM-EELS results and companion simulations indicate that increasing the Pd content in the NR increases the FWHM of the LSP modes. This behaviour indicates an increase in damping of the LSP modes, which is expected when alloying a plasmonic metal with a weakly plasmonic material. Yet, in spite of the increase in damping, Cu–CuPd NRs with Pd content of up to 46% by mass still support resolvable 
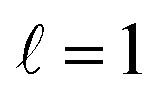
 LSP modes. We therefore conclude that Cu NRs can be alloyed with substantial amounts of Pd and still support plasmon resonances. This may be exploited to address Cu's environmental sensitivity and provide additional functionality. Moreover, alloying metal NPs with Pd is expected to be translatable to other metal NPs, paving the way for further multimetallic Pd nanostructures which can remain plasmonic.

## Author contributions

Conceptualisation: SJ and XY. Data curation: AT, CAW, SJ, and ERH. Formal analysis: AT. Funding acquisition: QMR, XY, and ER. Investigation: SJ, ERH, YW, BZ, QMR and ER. Methodology: CAW, SJ and XY. Project administration, resources, and supervision: XY and ER. Software: CAW. Visualisation and writing – original draft: AT and CAW. Writing – review & editing: AT, CAW, SJ, ERH, QMR, XY, and ER.

## Conflicts of interest

There are no conflicts to declare.

## Supplementary Material

NA-005-D3NA00523B-s001
