# Supplementary material for: Bimetallic copper palladium nanorods: plasmonic properties and palladium content effects
Source: Nanoscale Adv. 2023 Oct 24;5(23):6524–32. doi: 10.1039/d3na00523b (PMC10662198; doi:10.1039/d3na00523b)
Supplement: NA-005-D3NA00523B-s001 [file NA-005-D3NA00523B-s001.pdf]

Electronic Supplementary Material (ESI) for Nanoscale Advances.
This journal is © The Royal Society of Chemistry 2023

Electronic Supplementary Information (ESI) for

Bimetallic Copper Palladium Nanorods: Plasmonic Properties and Palladium Content Effects

Andrey Ten,[‡ab] Claire A. West,[‡ab] Soojin Jeong,[c] Elizabeth R. Hopper,[abd] Yi Wang,[c] Baixu Zhu,[c] Quentin M. Ramasse,[efg] Xingchen Ye*[c] and Emilie Ringe*[ab]

a. Department of Materials Science and Metallurgy, University of Cambridge, 27 Charles Babbage Road, Cambridge CB3 0FS, United Kingdom
b. Department of Earth Sciences, University of Cambridge, Downing Street, Cambridge CB2 3EQ, United Kingdom
c. Department of Chemistry, Indiana University, 800 East Kirkwood Avenue, Bloomington, Indiana 47405, United States of America
d. Department of Chemical Engineering and Biotechnology, University of Cambridge, Philippa Fawcett Drive, Cambridge CB3 0AS, United Kingdom
e. School of Chemical and Process Engineering, University of Leeds, Leeds LS2 9JT, United Kingdom
f. School of Physics and Astronomy, University of Leeds, Leeds LS2 9JS, United Kingdom
g. SuperSTEM, SciTech Daresbury Science and Innovation Campus, Keckwick Lane, Daresbury WA4 4AD, United Kingdom

‡These authors contributed equally to this work

*Corresponding authors: X.Y.: xingye@indiana.edu, E.R.: er407@cam.ac.uk

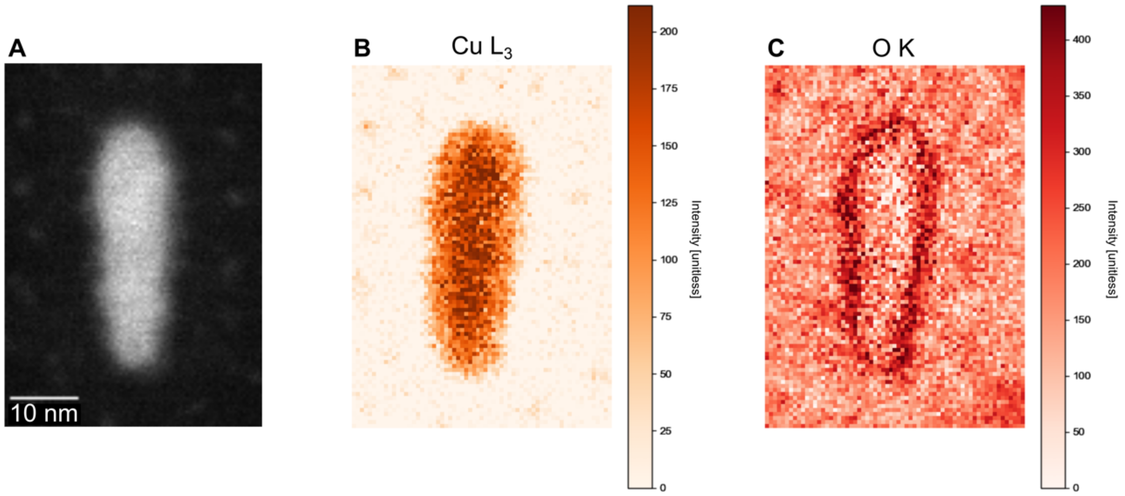

Figure S1. Core-loss EELS elemental maps of a Cu NR (36.4 nm x 11.8 nm). (A) HAADF-STEM image of the Cu NR, (B) map of Cu content, produced by integrating the Cu L_3-edge at 931 eV, (C) map of O content, produced by integrating the O K-edge at 530 eV.

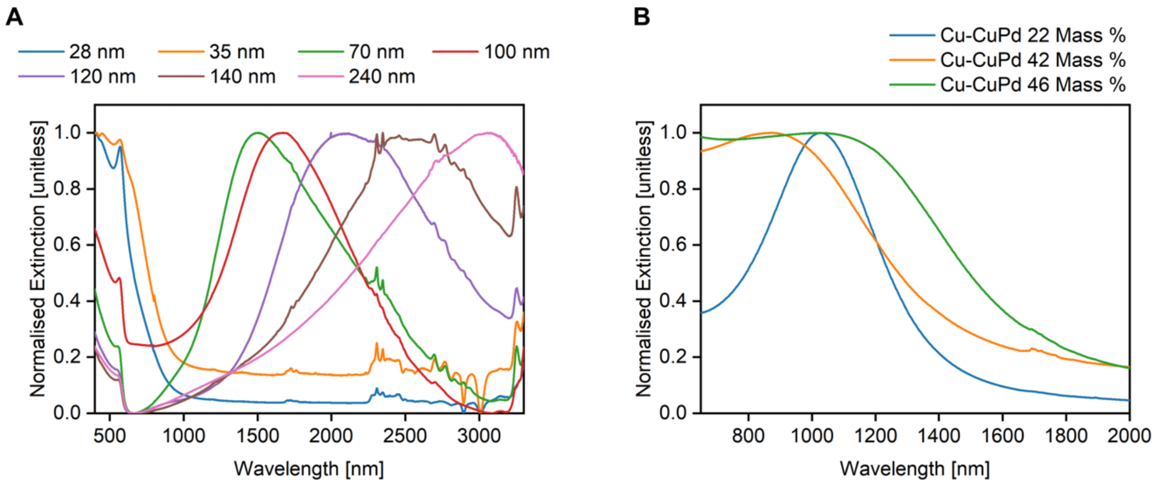

Figure S2. UV-Vis-NIR extinction spectra of (A) Cu NRs of labelled lengths and (B) Cu-CuPd NRs of labelled mass percents. The width of Cu NRs was constant at ~12 nm and LSPR peak for Cu NRs redshifted with increasing length. Cu-CuPd NRs did not show a trend in peak position, but the LSPR peak was found to broaden with increased Pd content.

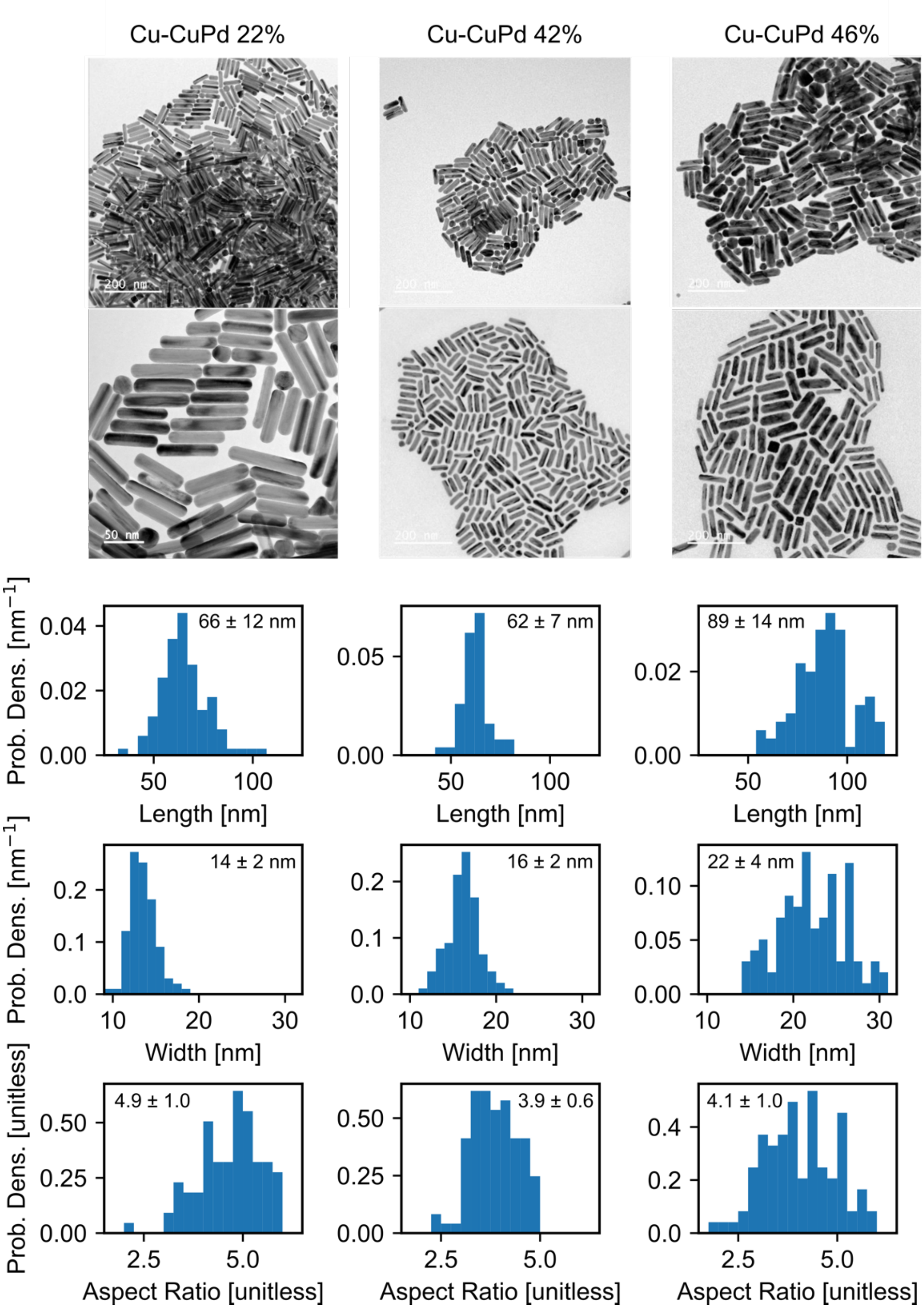

Figure S3. TEM images and length, width, and aspect ratio distributions of as-synthesised Cu-CuPd NRs with (left) 22, (middle) 42, and (right) 46% by mass Pd. 100 NRs were sampled from each set. Average and standard deviation are labelled for each distribution.

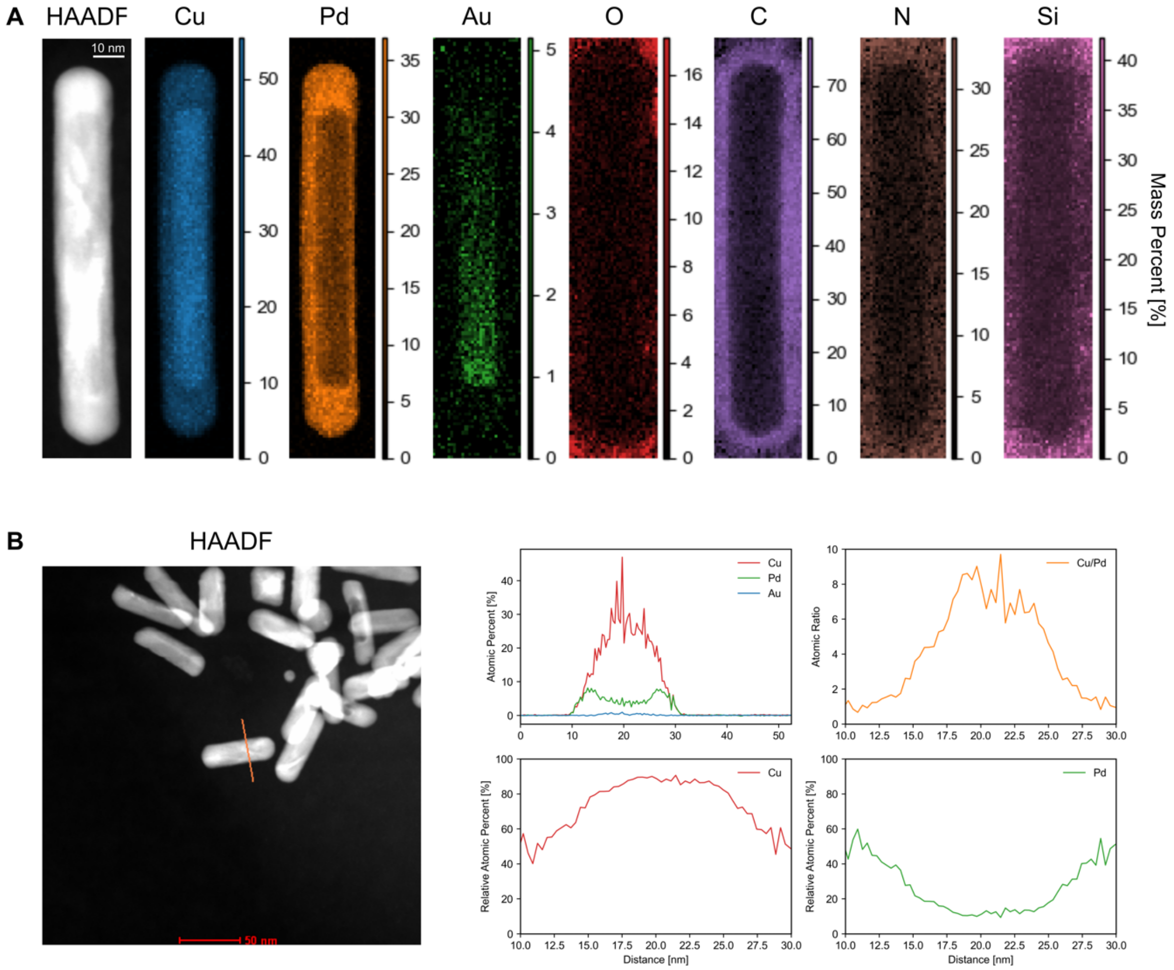

Figure S4. (A) HAADF-STEM and STEM-EDS elemental maps of the Cu-CuPd NR (46%) in Figure 1 of the main text, showing all atomic compositions in mass percent, quantified using the Cliff-Lorimer ratio method. The colour scale is indicative of the percent by mass of each atom. Si and N signals originate from the Si_3N_4 membrane substrate, while the C signal originates from organic contamination as well as C deposited around the NR during acquisition. (B) HAADF-STEM image and representative STEM-EDS line scan of the Cu-CuPd NR (42%) showing the atomic percentages of Cu, Pd, and Au, and relative atomic ratios between Cu and Pd.

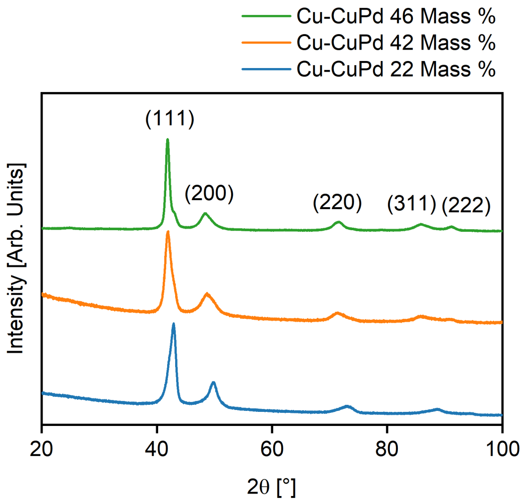

Figure S5. X-ray diffraction patterns of Cu-CuPd NRs with 22, 42, and 46% by mass Pd, showing the reflections of the CuPd disordered A1 phase which were assigned based on Ref. 65 of the main text.

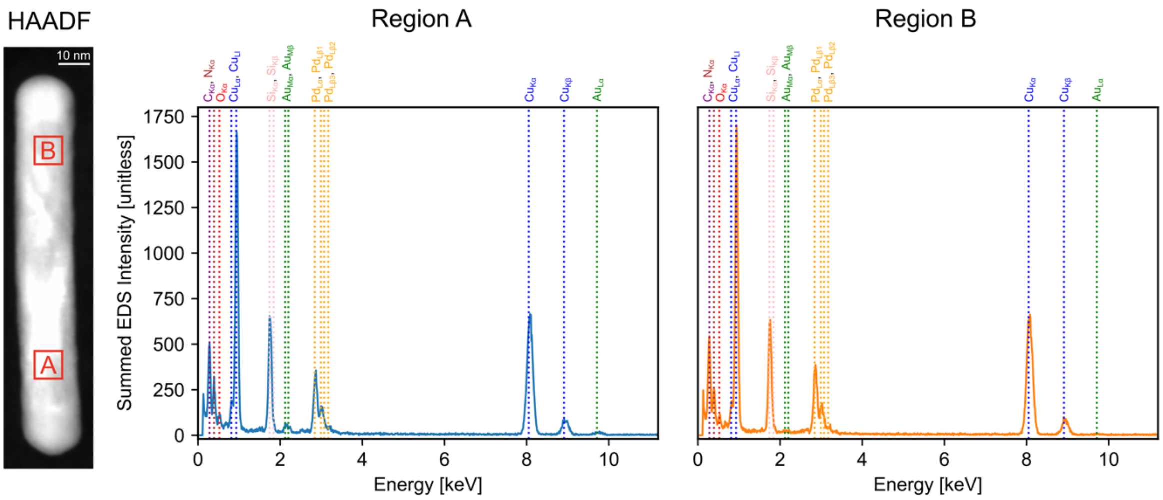

Figure S6. HAADF-STEM and STEM-EDS summed over 8 x 8 pixels as indicated by A and B around the tip of the Cu core in the Cu-CuPd NR (46%) in Figure 1 of the main text. Au L_{α} (9.71 keV), Au M_{α} (2.12 keV) and Au M_{β} (2.20 keV) lines are only present in region A.

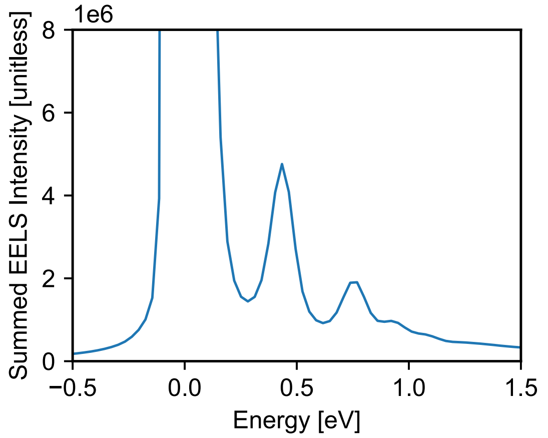

Figure S7. STEM-EEL spectrum summed across the spectrum image of the 270 nm x 12 nm Cu NR. The dipolar LSP mode is at 0.42 eV (confirmed by NMF analysis) and is well separated from the zero-loss peak (ZLP) at 0.0 eV. The substantial separation of modes, here and in other NRs, negated the need for any deconvolution from the ZLP.

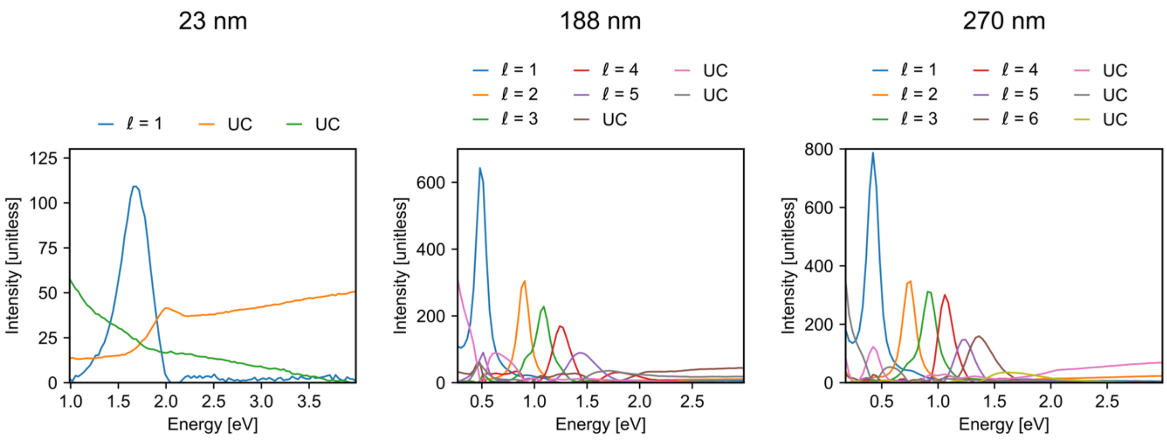

Figure S8. STEM-EELS NMF spectral components for the three representative Cu NRs of the labelled length and width of 12-13 nm (same NRs as in Figure 3A). LSP modes were assigned based on the peak energy and corresponding spatial loadings. There remained residual unassigned components (UC) from every NMF decomposition.

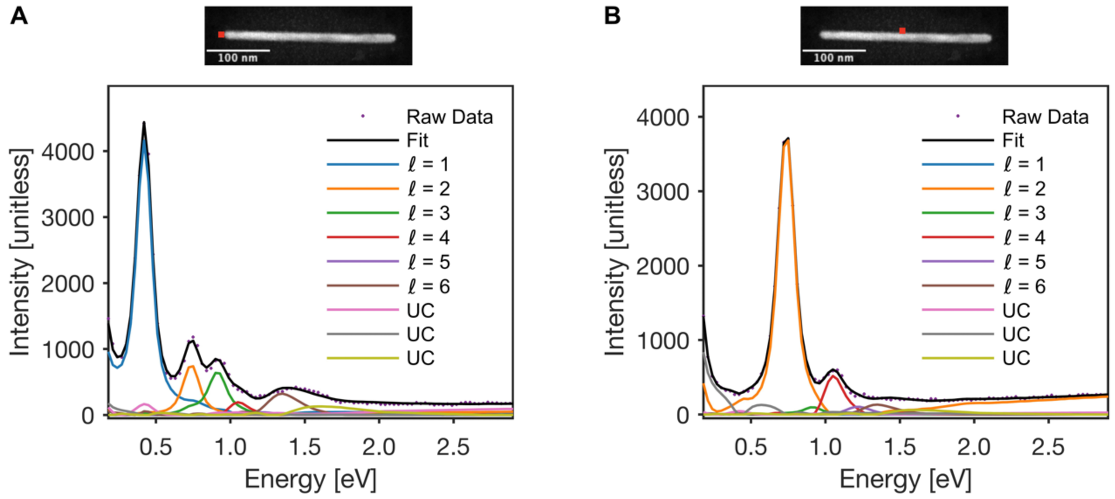

Figure S9. STEM-EELS NMF spectral components for the 270 nm Cu NR (same NR as in Figure 3A) fitted to the 3 x 3 pixels in the region indicated by the red squares in the HAADF-STEM images of (A) and (B).

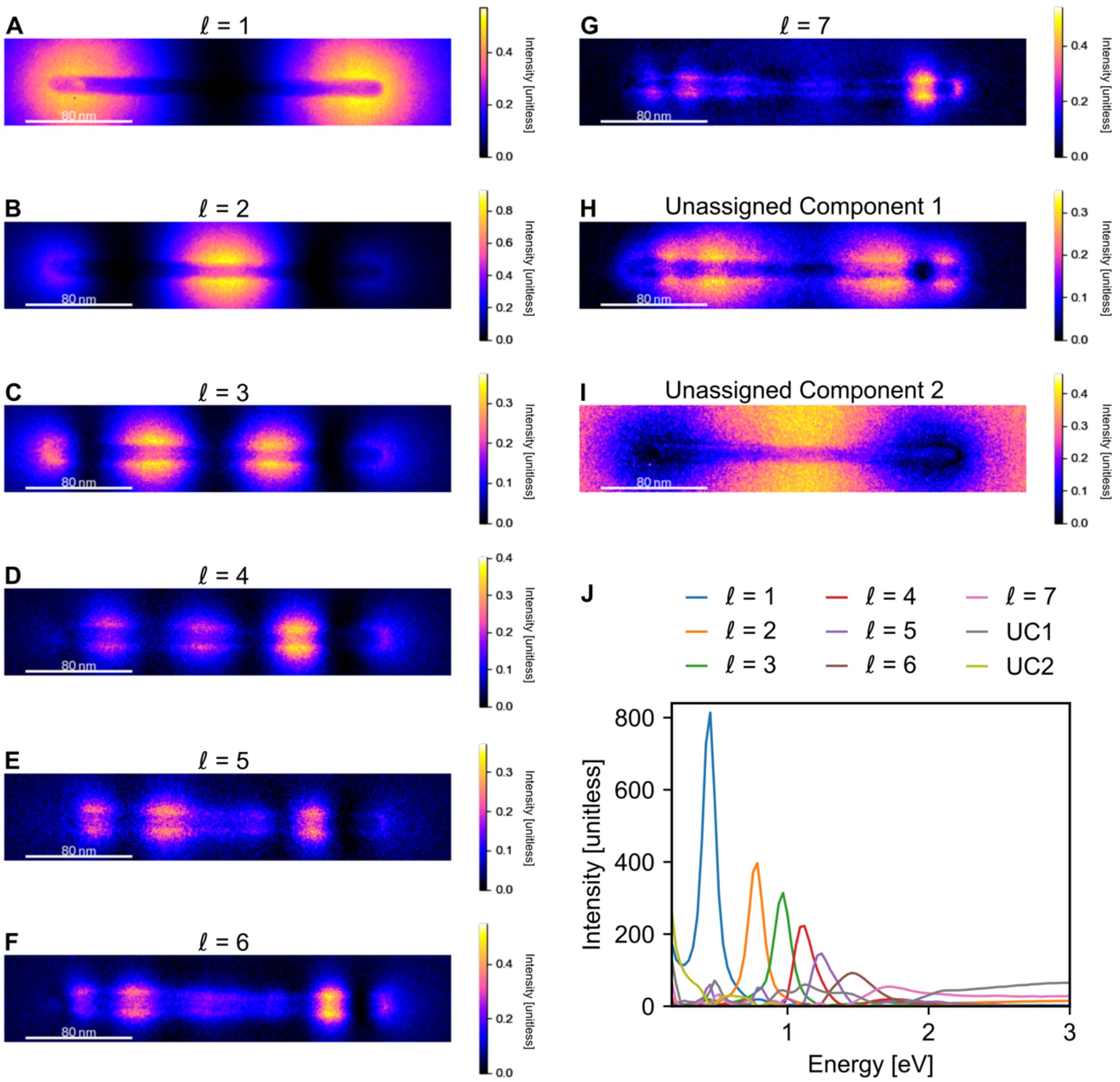

Figure S10. STEM-EELS NMF spatial loadings (A-I) and their spectral components (J) for the 258 nm x 11 nm Cu NR, showing up to seven resolvable modes. There remained two residual unassigned components (UC1 and UC2) from this NMF decomposition.

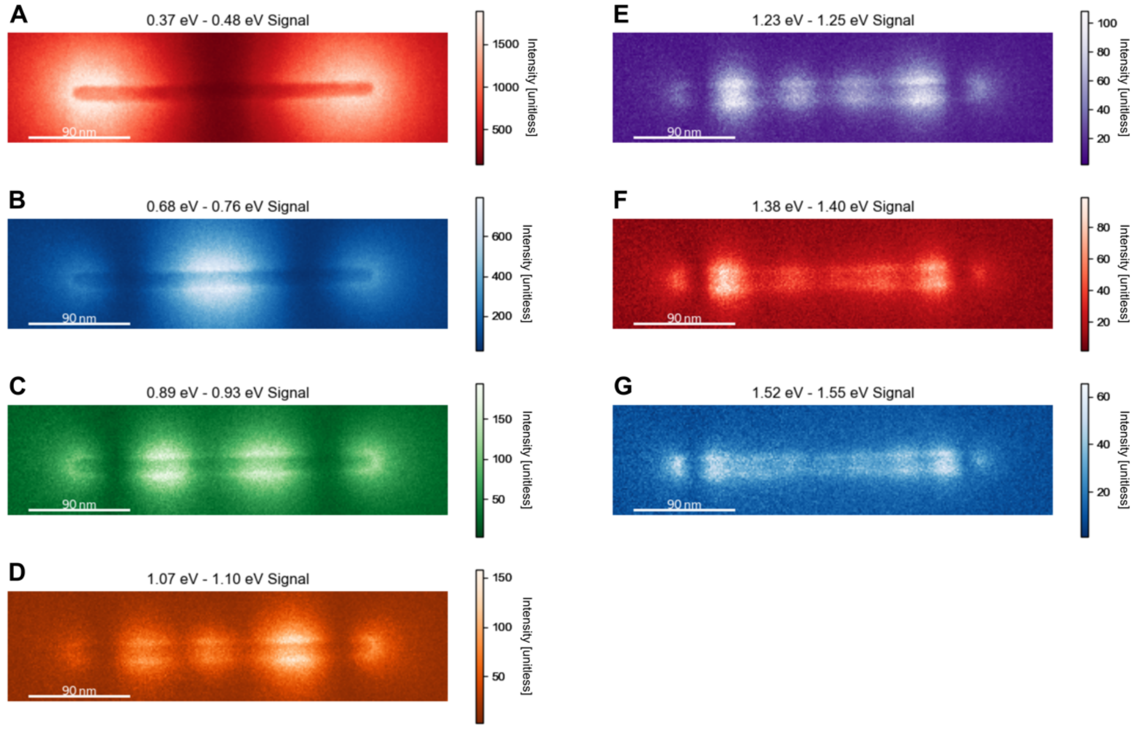

Figure S11. Energy slices extracted from the STEM-EELS data set of the 270 nm Cu NR (same NR as in Figure 3A) in the labelled range of energy loss.

Table S1. Dimensions of Cu NRs used for STEM-EELS analysis.

Length (nm)	Width (nm)	Aspect Ratio	Length (nm)	Width (nm)	Aspect Ratio
22.5	13.3	1.7	137.5	10.9	12.6
25.7	14.3	1.8	146.6	11.0	13.4
33.1	11.7	2.8	149.0	11.2	13.3
35.5	9.7	3.7	171.9	11.9	14.5
45.6	12.4	3.7	173.7	8.9	19.6
59.1	8.6	6.9	187.2	12.8	14.7
61.7	10.5	5.9	189.1	11.7	16.1
79.0	10.0	7.9	207.6	12.5	16.6

80.2	13.7	5.9	208.3	11.7	17.8
82.0	9.9	8.3	225.8	11.1	20.3
91.0	6.9	13.1	241.2	11.1	21.8
100.0	12.3	8.1	257.2	12.7	20.3
105.5	12.1	8.7	258.2	11.2	23.1
112.9	10.5	10.7	261.4	12.5	20.9
117.7	9.9	11.9	268.4	12.5	21.5
126.6	10.9	11.6	270.0	12.3	21.9
136.2	12.0	11.4	399.0	12.5	31.9
136.7	11.1	12.4			

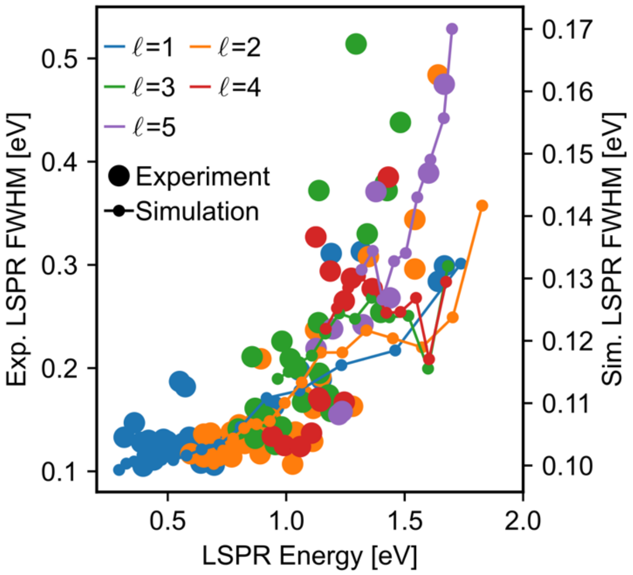

Figure S12. Experimental and simulated LSPR FWHM as a function of resonance energy for modes $\ell = 1 - 5$.

Table S2. Dimensions of Cu-CuPd NRs used for STEM-EELS analysis.

Pd Content	Length (nm)	Width (nm)	Aspect Ratio
22%	49.0	22.8	2.2
	64.0	16.2	3.9
	64.9	14.3	4.5
	66.8	15.8	4.2
	72.9	14.8	4.9
	77.1	13.3	5.8
	85.4	16.0	5.3
	88.0	15.9	5.5
	90.1	16.4	5.5
42%	47.3	21.7	2.2
	57.0	19.1	3.0
	60.3	18.0	3.4
	62.1	17.3	3.6
	69.5	18.0	3.9
46%	60.9	28.1	2.2
	70.1	18.0	3.9
	71.1	16.8	4.2
	74.9	16.4	4.6
	75.3	18.9	4.0
	86.3	27.5	3.1
	93.8	14.5	6.5
	102.8	18.5	5.6

	108.4	23.8	4.5
	109.4	19.3	5.7

Table S3. STEM-EELS signal intensity of the dipole peak in relation to the ZLP for Cu and Cu-CuPd NRs of various Pd content (22, 42, and 46%). Three NRs from each batch were selected at random.

	Dipole peak intensity divided by the ZLP intensity (%)
Cu NRs	0.186
	0.218
	0.100
Cu-CuPd NRs (22%)	0.070
	0.057
	0.049
Cu-CuPd NRs (42%)	0.041
	0.035
	0.039
Cu-CuPd NRs (46%)	0.089
	0.077
	0.045

Mitigations made to STEM-EELS analysis for Cu-CuPd NRs to eliminate artefacts caused during acquisition by C contamination

The STEM-EELS spectrum images were acquired individually for each NR. In each case, the NR was scanned along the short axis (perpendicular to the rod axis) before being scanned for the next row along the long axis (parallel to the rod axis, see Figure S13). Over time, each sample experienced considerable C contamination. Thus, data acquired at the later stage of scanning probed an increasingly more contaminated NR. C contamination was difficult to avoid as NRs were capped with OLAM and grafted with PS-PEHA for colloidal stability. Neither baking nor plasma cleaning were applied as they posed a risk of oxidising the NRs.

The effect of C contamination is observed through various parameters. First, we find that LSPR energies of the part of the NR scanned later was redshifted compared to the earlier scans of the same NR (Figure S13), consistent with a dielectric environment with added C

around the NR. We also observed a weak broadening of the LSPR linewidth at later scans (Figure S13). Lastly, the HAADF images taken after the EELS scan showed lower contrast compared to the images taken before the scan. This arises from the fact that the extra carbon layer becomes the entrance plane for the electrons during the scan. This causes the beam to broaden while it travels through the carbon, and by the time it reaches the NR, the imaging probe is 'blurred,' resulting in less contrast on the actual object of interest. One might wonder why the additional scattering from deposited C does not increase the contrast in HAADF images; this is because C scatters weakly and at medium angles rather than into the HAADF detector.

The NMF analysis involves a global fit to the spectrum image, and with LSPR energies being redshifted in the part of the image scanned later, the linewidths of each mode were broadened by the shifted amount. For Cu NRs, C deposition, and thus this shift, was insignificant and did not influence the NMF analysis, apart from the negligible systematic increase on the FWHM of LSP modes. Cu-CuPd NRs experienced more significant shifts to their LSPR energies, possibly due to the presence of leftover Pd precursors. For most Cu-CuPd NRs, NMF factorisation identified parts of the same mode (mainly the dipolar mode) as individual factors: one factor containing the dipolar mode in the part of the image acquired first, and another containing the part acquired towards the end of the scan. Decreasing the number of decomposition factors to combine both parts of the mode into a single factor was possible and the NMF spatial loadings (Figure 5B) were extracted using this method. However, extracting LSPR energies and FWHM from the combined spectral factors was misleading, as the resulting LSPR energies were averaged, and linewidth broadened. As such, a different approach was taken for the extraction of LSPR energies and their FWHM. We cropped the spectrum image in half, perpendicular to the rod axis, to contain only half of the NR that was scanned first. EELS point spectra (Figure 5A) were extracted near the tip of the NR in this cropped image. NMF decomposition was performed on this half of the image to extract LSPR energies and corresponding FWHMs (Figures 6A and 6B).

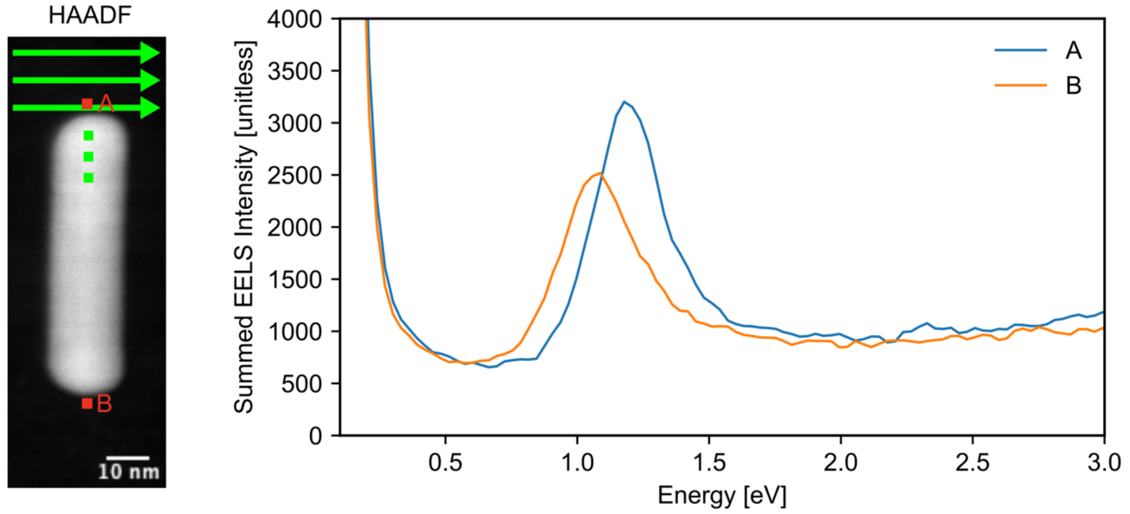

Figure S13. Effect of C contamination over an EELS scan of a Cu-CuPd NR (22%, 64 nm x 16 nm). The green arrows on the HAADF-STEM image show the direction of an EELS scan, and after each horizontal scan the beam is moved down for the next row of pixels (arrows bigger than the actual scan width). EEL point spectra summed over 5 x 5 pixels in regions A and B, showing the dipolar LSPR peak. The dipolar LSPR peak appears redshifted and broadened in the region scanned last (B) compared to the region scanned first (A). The peak intensity differences between spectra from A and B are not directly comparable as the integrated areas do not overlap equivalently with the plasmon excitation distribution.
